# Adropin-based dual treatment enhances the therapeutic potential of mesenchymal stem cells in rat myocardial infarction

**DOI:** 10.1038/s41419-021-03610-1

**Published:** 2021-05-18

**Authors:** HuiYa Li, DanQing Hu, Guilin Chen, DeDong Zheng, ShuMei Li, YunLing Lin, HuaShan Hong, Yukun Luo, YiLang Ke, Yu Huang, LingZhen Wu, TingXiang Lan, WenYing Wang, Jun Fang

**Affiliations:** 1 Department of Cardiology, Fujian Institute of Coronary Heart Disease, Fujian Heart Medical Center, Fujian Medical University Union Hospital, Fuzhou, PR China; 2 YinZhou People’s Hospital & Affiliated Hospital, Medical School, Ningbo University, Ningbo, PR China; 3 Department of Pharmacology, School of Basic Medical Sciences, Tianjin Medical University, Tianjin, PR China; 4 Department of Emergency, People’s Hospital of Longhua, Shenzhen, PR China; 5 Department of Geriatrics, Fujian Key Laboratory of Vascular Aging, Fujian Institute of Geriatrics, Fujian Medical University Union Hospital, Fuzhou, PR China

**Keywords:** Cell death, Heart stem cells

## Abstract

Both weak survival ability of stem cells and hostile microenvironment are dual dilemma for cell therapy. Adropin, a bioactive substance, has been demonstrated to be cytoprotective. We therefore hypothesized that adropin may produce dual protective effects on the therapeutic potential of stem cells in myocardial infarction by employing an adropin-based dual treatment of promoting stem cell survival in vitro and modifying microenvironment in vivo. In the current study, adropin (25 ng/ml) in vitro reduced hydrogen peroxide-induced apoptosis in rat bone marrow mesenchymal stem cells (MSCs) and improved MSCs survival with increased phosphorylation of Akt and extracellular regulated protein kinases (ERK) l/2. Adropin-induced cytoprotection was blocked by the inhibitors of Akt and ERK1/2. The left main coronary artery of rats was ligated for 3 or 28 days to induce myocardial infarction. Bromodeoxyuridine (BrdU)-labeled MSCs, which were in vitro pretreated with adropin, were in vivo intramyocardially injected after ischemia, following an intravenous injection of 0.2 mg/kg adropin (dual treatment). Compared with MSCs transplantation alone, the dual treatment with adropin reported a higher level of interleukin-10, a lower level of tumor necrosis factor-α and interleukin-1β in plasma at day 3, and higher left ventricular ejection fraction and expression of paracrine factors at day 28, with less myocardial fibrosis and higher capillary density, and produced more surviving BrdU-positive cells at day 3 and 28. In conclusion, our data evidence that adropin-based dual treatment may enhance the therapeutic potential of MSCs to repair myocardium through paracrine mechanism via the pro-survival pathways.

## Introduction

Stem cell therapy to repair infarcted myocardium is generally established as a promising strategy to treat heart failure, and the paracrine effects may be a more important mechanism than myocardial regeneration^1–4^.

The success of the cell therapy has been compromised by a dual dilemma of the low survival rate of donor cells (the “seed”) and hostile host microenvironment (the “soil”)^1,4–9^. Obviously, to achieve ideal therapeutic efficiency, stem cell therapy should seek a new strategy that adopts a synergistic manner to target the “seed” to improve the survival potential and the “soil” to modify the hostile microenvironment in myocardial infarction (MI)^8,10^. However, the research on dual protective strategies for stem cell therapy remains limited.

Adropin, a secreted protein encoded by the energy homeostasis-associated gene^11^, has been identified as a novel endogenous bioactive factor to protect endothelial function through the pro-survival reperfusion injury salvage kinase (RISK) pathway^12^, which consists of phosphatidylinositol 3-kinase (PI3K)/Akt and extracellular regulated protein kinases (ERK) 1/2 pathways^13^. Recently, we reported that adropin may offer in vitro protection against hypoxia/reoxygenation-induced cardiomyocyte injury by suppressing apoptosis, inflammation, and oxidative stress through the RISK pathway^14^.

In this study, on the basis of the potential cytoprotective effects by activating pro-survival kinases in multiple cell types, adropin was in vitro used to improve the survival potential of mesenchymal stem cells (MSCs), and meanwhile, in vivo to attenuate the inflammation in rat MI. Then, the adropin-treated MSCs (modified “seed”) were transplanted into the adropin-treated injured myocardium (modified “soil”), thus implementing dual protective effects to enhance the therapeutic potential of MSCs via the paracrine mechanism. We also in vitro tested whether adropin may attenuate hydrogen peroxide (H_2_O_2_)-induced apoptosis of MSCs through the pro-survival kinase pathway. This may represent an optimized strategy to improve the feasibility of MSCs transplantation in MI treatment.

## Methods

The study was in compliance with the Guid*e for the Care and Use of Laboratory Animals* published by the US National Institute of Health (8th Edition, 2011) and was approved by the Experimental Animal Care Committee of Fujian Medical University Union Hospital.

### Isolation, culture, identification, and labeling of rat bone marrow MSCs

In this study, male Sprague-Dawley (SD) rats weighing (100 ± 10) g (Shanghai Slac Laboratory Animal Co. Ltd., China) were used to isolate bone marrow MSCs as described previously^4^. Flow cytometry (FACScan, Becton-Dickinson, USA) was used to detect the superficial markers of MSCs, including the cells positive for CD44 (Biolegend), CD90 (Biolegend), and CD29 (Biolegend) and negative for CD45 (Biolegend), CD34 (Santa), and CD11b (Biolegend)^4^. MSCs at passage 3 were labeled with bromodeoxyuridine (BrdU) (Sigma, USA) 48 h at a final concentration of 10 μmol/L, and the efficiency of labeling was evaluated under a laser scanning confocal microscope (LSM510, Zeiss) equipped with blue argon (for 4′,6-diamidino-2-phenylindole, DAPI) and red krypton (for BrdU) lasers. Before transplantation, BrdU-labeled MSCs were pretreated with adropin for 24 h at a final concentration of 25 ng/ml.

### In vitro H_2_O_2_-induced apoptosis of rat bone marrow MSCs

Different final concentrations of H_2_O_2_ (0, 50, 100, and 200 μmol/L) (Aladdin), characterized by oxidative damage induction^15^, were used to induce MSCs apoptosis for the indicated periods (0, 1, 2, and 4 h), and a final concentration of 100 μmol/L for 4 h was used to investigate the protective effect of adropin against MSCs damage (Supplementary Fig. 1). Except those of the control group (Control) cultured under normal condition, other MSCs underwent H_2_O_2_ treatment and were divided into six groups: (1) the apoptosis model group (H_2_O_2_): treated with 100 μmol/L H_2_O_2_ for 4 h; (2)–(4) the low-, moderate-, and high-dose adropin groups (Ad-L, Ad-M, and Ad-H, respectively): treated like the apoptosis model group, but pretreated with 10, 25, and 50 ng/ml adropin (Phoenix Pharmaceuticals, USA) for 24 h, respectively; (5) the LY294002 + Ad-M group (LY): treated like the Ad-M but pretreated with PI3K specific inhibitor LY294002 (Sigma, USA) at 50 μmol/L for 2 h; (6) the PD98059 + Ad-M group (PD): treated like the Ad-M but pretreated with ERK1/2 specific inhibitor PD98059 at 50 μmol/L for 30 min. Cell viability (survival) was assessed by Cell Counting Kit-8 (CCK-8), apoptosis rate by flow cytometry, and the protein expression of Akt, ERK1/2, and antiapoptotic proteins [B-cell lymphoma-2 (BCL-2) and B-cell lymphoma extra-large (BCL-XL)] by western blotting.

### Cell viability assessment by CCK-8

MSCs from various groups were seeded into 96-well plates at the same density and cultured. At the end of each culture, the medium was removed and replaced with 100 μl of fresh medium and 10 μl of CCK-8 reagent (Phygene life science, Shanghai, China). Plates were incubated at 37 °C for 1.5 h, and then absorbance was measured at 450 nm using a SpectraMax 190 microplate reader (Molecular Devices). The reference wavelength was 600 nm. Background absorbance (from wells without cells) was subtracted from all values.

### Flow cytometric analyses of MSCs apoptosis

An Annexin-V-FLUOS Staining Kit (Roche) was used to quantitatively determine the percentage of cells undergoing apoptosis and survival according to the manufacturer’s instructions. Briefly, MSCs were collected and resuspended in the binding buffer. Annexin-V-FITC and propidium iodide (PI) were added to the suspended cells, and the reaction was incubated in the dark for 10 min. The percentage of apoptotic cells was analyzed by flow cytometry (FACScan, Becton-Dickinson). The experiment was repeated three times.

### In vivo experimental protocol

A total of 64 male SD rats (weighed 200 ± 50 g), were enrolled in the experiment. The left main coronary arteries (LCA) of rats were occluded for 3 days or 28 days to establish a MI model. All rats were randomly divided into four groups (*n* = 16 per group): the Sham group, with a silk suture placed under the left main coronary artery but not ligated, and the same solvents of MSCs and adropin intramyocardially and intravenously injected, respectively; the MI group, with the same solvents of MSCs and adropin intramyocardially and intravenously injected, respectively, and without any other intervention; the MI + MSC group, treated like the MI group but given an intramyocardial injection of BrdU-MSCs at 10 min after ischemia and an intravenous injection of the same solvents of adropin; the MI + MSC + Adropin group, treated like the MI group but given an intramyocardial injection of adropin-pretreated BrdU-MSCs, following an injection of 0.2 mg/kg adropin via tail veins at 10 min after ischemia.

The choice of the adropin dose (0.2 mg/kg) was based on an additional dose-effect cardioprotective study (Supplementary Fig. 2), which used a rodent model of myocardial ischemia-reperfusion injury. We enrolled a total of 30 wild-type C57BL/6 mice (sex in half), aged 20 weeks old and weighed 25–35 g (Shanghai SLAC laboratory Animal Co., Ltd., China). Except those of the Sham group (Sham) without ischemia (*n* = 6, sex in half), other mice underwent ischemia (I) for 45 min followed by reperfusion (R) for 4 h, and were assigned to four groups (*n* = 6, sex in half): the I/R group: treated with ischemia followed by reperfusion; the low-, moderate-, and high-dose adropin groups (Adropin-L, Adropin-M, and Adropin-H, respectively): receiving the same treatment as I/R and a jugular venous injection of 0.1, 0.2, and 0.4 mg/kg adropin (Phoenix Pharmaceuticals, USA), respectively, 5 min before reperfusion.

### Surgical preparation and MSCs transplantation

To establish the in vivo model of MI, rats were intraperitoneally sedated with 75 mg/kg ketamine and 7.5 mg/kg diazepam before surgery and ventilated by a rodent ventilator (tidal volume, 1.0 ml/100 g body weight; respiratory rate, 50–60 cycles per minute)^4^. The chest was opened via a left thoracotomy through the fourth intercostal space to expose the heart. After pericardiotomy, the left main coronary artery was ligated with a silk 8-0 suture for 3 d or 28 d to induce MI^4^. In the MSCs transplantation groups, the ischemic region received intramyocardially at four sites a total of 50 μl BrdU-labeled MSCs (1 × 10^6^ cells). The groups without cell therapy received the same volume of medium. At day 3 or day 28 of MI, rats (*n* = 8 for each time point in each group) were sacrificed by an intravenous bolus injection of 10% KCl under deep anesthesia, following venous blood sample collection and echocardiographic assessment. The heart tissues were stored at −80 °C for histological studies and assessment of vascular endothelial and basic fibroblast growth factor (VEGF and bFGF) by real-time (RT)-PCR and western blotting.

Surgical preparations of mice were adapted from procedures previously described^4,16^. After pericardiotomy, silk 8-0 sutures were placed under the left main coronary artery, and the ends of the ties were threaded through small polyethylene tubes to form snares for reversible coronary artery occlusion. At 4 h R, to assess the area at risk, the left ventricle at risk was determined by injecting 1 ml of 0.1% Evan’s blue (Sigma, USA) after the left main coronary artery was ligated again. The heart was harvested and cut transversely into three slices parallel to the atrioventricular groove at a thickness of 1–2 mm for triphenyltetrazolium chloride staining, and the area at risk and infarct size were determined^17^.

### Echocardiographic examination

Cardiac function was assessed by transthoracic echocardiography at day 3 and day 28 after cell transplantation. Short-axis two-dimensional view of the left ventricle was taken at the level of the papillary muscles to obtain the M-mode recordings with a 10 MHz electronic-phased-array transducer (GE Vingmed Ultrasound VIVID7, USA). The left ventricular end-systolic diameter (ESd) and end-diastolic diameter (EDd) were measured from at least three consecutive cardiac cycles. Fractional shortening (FS) (%) was calculated as (EDd − ESd)/EDd × 100; end-diastolic volume (EDv) as 7.0 × EDd3/(2.4 + EDd); end-systolic volume (ESv) as 7.0 × ESd3/(2.4 + ESd); and ejection fraction (EF) (%) as (EDv − ESv)/EDv × 100^4^.

### Histological studies

Immunohistochemical staining was used to detect the surviving BrdU-positive cells at day 3 and 28, and capillary regeneration at day 28 after cell transplantation^4^. After blockage of endogenous peroxidase activity by 3% H_2_O_2_, antigen retrieval with sodium citrate, and blockage of nonspecific immunoreactivity by normal goat serum, paraffin sections (4 μm) were incubated with anti-BrdU antigen (Sigma) and anti-factor VIII-related antigen (Dako, Denmark) and horseradish peroxidase-conjugated second antibodies. Color development in immunoperoxidase staining was performed with diaminobenzidine and sections were counterstained using hematoxylin. To quantify capillary regeneration, three sections from each heart were selected for analysis and five fields from each section were randomly counted. Capillary density was quantified as the number of factor VIII-related antigen-positive vessels (diameter <10 μm) per high-power field (0.06 mm^2^). Surviving transplanted MSCs were quantified as the number of BrdU-positive cells per 1 mm^2^ field. Myocardial fibrosis at day 28 was observed by collagen-specific Masson’s trichrome staining. The quantification analysis was performed by two observers blinded to the treatment of the animals.

### Western blotting

The protein expression of phosphor (p)-Akt, total (t)-Akt, p-ERK1/2, t-ERK1/2, BCL-2, BCL-XL, VEGF, and bFGF was analyzed by western blotting. Protein extract was loaded onto a polyacrylamide gel used for electrophoresis. Then, the protein was transferred onto a polyvinylidene fluoride membrane. The membrane was incubated in TBST containing 5% nonfat dry milk for 1 h at room temperature. The membranes were then incubated with antibodies of Akt, p-Akt, ERK1/2, p- ERK1/2, BCL-2, BCL-XL, VEGF and bFGF (all from CST, USA), or β-actin antibody (EMAR BIO) at 4 °C overnight followed by secondary antibody for 1 h at room temperature. The ratios of p-Akt/t-Akt and p-ERK/t-ERK were calculated, and glyceraldehyde-3-phosphate dehydrogenase (GAPDH) was used as loading control. Target signals of BCL-2, BCL-XL, VEGF, and bFGF were normalized relative to the β-actin expression.

### Quantitative real-time PCR

The gene expression of VEGF and bFGF at day 28 after cell transplantation was detected by real-time PCR (IQ5, Bio-Rad, USA). The extraction of total RNA from heart tissues and their subsequent first-strand cDNA synthesis were performed with a TRIzol reagent (GIBCO/BRL, USA) and a M-MLV Reverse Transcriptase kit (Promega, USA), respectively. The following primer sequences were used: VEGF, 5′-GCAATGATGAAGCCCTGGAG-3′ (F), 5′-CGCTCCAGGATTTAAACCGG-3′ (R); bFGF, 5′-GAACCGGTACCTGGCTATGA-3′ (F), 5′-CAGTTCGTTTCAGTGCCACA-3′ (R); GAPDH, 5′-CACTAAAGGGCATCCTGGGCTACAC-3′ (F), 5′-GGAGGCCATGTAGGCCATGAGG-3′ (R). After the activation of the AmpliTaq Gold (Applied Biosystems) for 2 min at 95 °C, 40 cycles were carried out with each cycle consisting of 10 s at 94 °C followed by 30 s at 60 °C. The dissociation curve for each amplification was analyzed to confirm that there were no nonspecific PCR products. All cDNA samples were amplified in triplicate and normalized against a triplicate of GAPDH in the same plate. The data were expressed as 2^−ΔΔCt^.

### ELISA

The concentration of rat plasma pro-inflammatory cytokines, interleukin (IL)-1β, tumor necrosis factor (TNF)-α, and anti-inflammatory cytokine IL-10 at 3 d MI was quantitatively determined with quantitative ELISA kits (R&D Systems, USA) according to the manufacturer’s instructions in a blinded manner. Briefly, antibodies specific for rat cytokines were pre-coated onto microplates. Standards, Control, and samples were pipetted into the wells and any rat cytokine present was bound by the immobilized antibody. After the removal of any unbound substances, an enzyme-linked polyclonal antibody specific for rat cytokine was added to the wells. After a wash to remove any unbound antibody-enzyme reagent, a substrate solution was added to the wells. The enzyme reaction yielded a blue product that turned yellow when the stop solution was added. The intensity of the color measured was in proportion to the amount of rat cytokine bound in the initial step. The sample values were then read off the standard curve.

### Statistical analysis

All data were presented as mean ± SD. Statistical analysis was performed with SPSS 17.0 for Windows. For comparisons between multiple groups, data were analyzed by ANOVA. When a statistical difference appeared, the least significant difference procedure was applied. A value of *p* < 0.05 was considered statistically significant.

## Results

### Adropin in vitro attenuates the apoptosis of oxidative stress-injured bone marrow MSCs

The characterization of cultured bone marrow MSCs was shown in Supplementary Fig. 3. About 87% of MSCs were successfully BrdU-labeled (Supplementary Fig. 4).

Various doses of adropin were used to investigate its effects on the H_2_O_2_-induced apoptosis of MSCs. Compared with that of the H_2_O_2_ group, the adropin pretreatment significantly increased the MSCs viability of the H_2_O_2_ + Ad-L group, the H_2_O_2_ + Ad-M group, and the H_2_O_2_ + Ad-H group in a dose-dependent manner (47.0 ± 2.0% vs. 63.6 ± 1.1%, 74.7 ± 3.8%, 78.3 ± 3.3%, *p* < 0.05, respectively), without any difference between the H_2_O_2_ + Ad-M (25 ng/ml) and H_2_O_2_ + Ad-H (50 ng/ml) groups (Supplementary Fig. 5A). Thus, the moderate dose (25 ng/ml) was selected for subsequent experiments. The H_2_O_2_ + Ad-M group exhibited an evidently lower early apoptosis rate than the H_2_O_2_ group (8.17% ± 1.65% vs. 40.63% ± 4.05%, *p* < 0.01, respectively), which indicates notable antiapoptotic effects of adropin on H_2_O_2_-induced oxidative stress damage of MSCs in vitro (Supplementary Fig. 5B, C).

### Adropin in vitro exerts antiapoptotic effects by activating the RISK pathways in oxidative stress-injured bone marrow MSCs

**Fig. 1:**
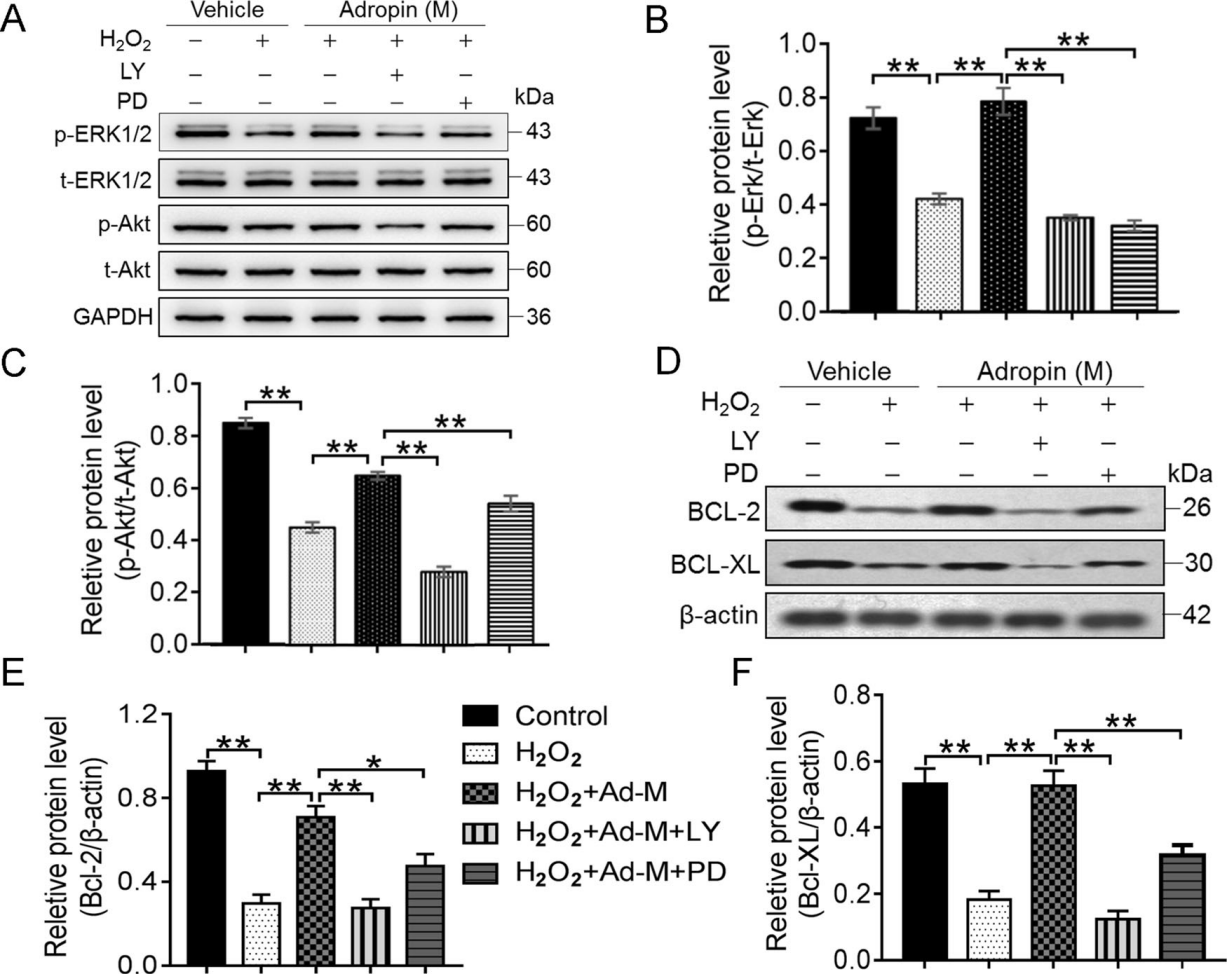
In vitro antiapoptotic effects on H_2_O_2_-induced apoptosis of bone marrow MSCs by adropin through activating the PI3K/Akt and ERK1/2 pathways. The antiapoptotic proteins BCL-2 and BCL-XL, and both total and phosphorylation levels of Akt and ERK1/2 were assessed in H_2_O_2_-injured MSCs by western blotting. Typical graphs of western blotting were shown in (**A**, **D**). **B**, **C**, **E**, **F** Quantitative western blotting analysis. MSCs Mesenchymal stem cells. H_2_O_2_ Hydrogen peroxide. Ad-M Moderate dose of adropin (25 ng/ml). LY LY294002. PD PD98059. p- Phosphorylation. t- Total. ERK1/2 Extracellular regulated protein kinase 1/2. PI3K Phosphatidylinositol 3-kinase. BCL-2 B-cell lymphoma-2. BCL-XL B-cell lymphoma-extra large. *p < 0.05; **p < 0.01.

We further explored whether the molecular mechanism underlying the protective effects of adropin on oxidative stress-injured MSCs involves the activation of PI3K/Akt and ERK1/2 signaling pathways, which were called the RISK pathway. The results demonstrated that adropin-induced antiapoptotic and pro-survival effects were blocked by the LY294002 (PI3K/Akt specific inhibitor) and PD98059 (ERK1/2 specific inhibitor), respectively (Supplementary Fig. 5D–F). H_2_O_2_ treatment inhibited the phosphorylation of Akt and ERK1/2 (p-Akt and p-ERK1/2), which was significantly reversed by adropin treatment, while no significant difference was found in total Akt and ERK1/2 (t-Akt and t-ERK1/2) (Fig. 1A–C). In addition, the increased activation of p-Akt and p-ERK1/2 by adropin was almost abolished by LY294002 and PD98059, respectively (Fig. 1A–C). The same results were observed in the protein expression of BCL-2 and BCL-XL (Fig. 1D–F).

These results indicate that adropin exerts antiapoptotic and pro-survival effects on oxidative stress-injured MSCs by activating the RISK pathway.

### Adropin-based dual treatment improves cardiac remodeling and function in rats by promoting the survival of transplanted MSCs and improving the host MI microenvironment

Since adropin may in vitro exert antiapoptotic and pro-survival effects on MSCs, we further evaluated the adropin-induced dual beneficial effects on cardiac functional recovery by modifying both transplanted MSCs (“seed”) and host MI microenvironment (“soil”).

**Fig. 2:**
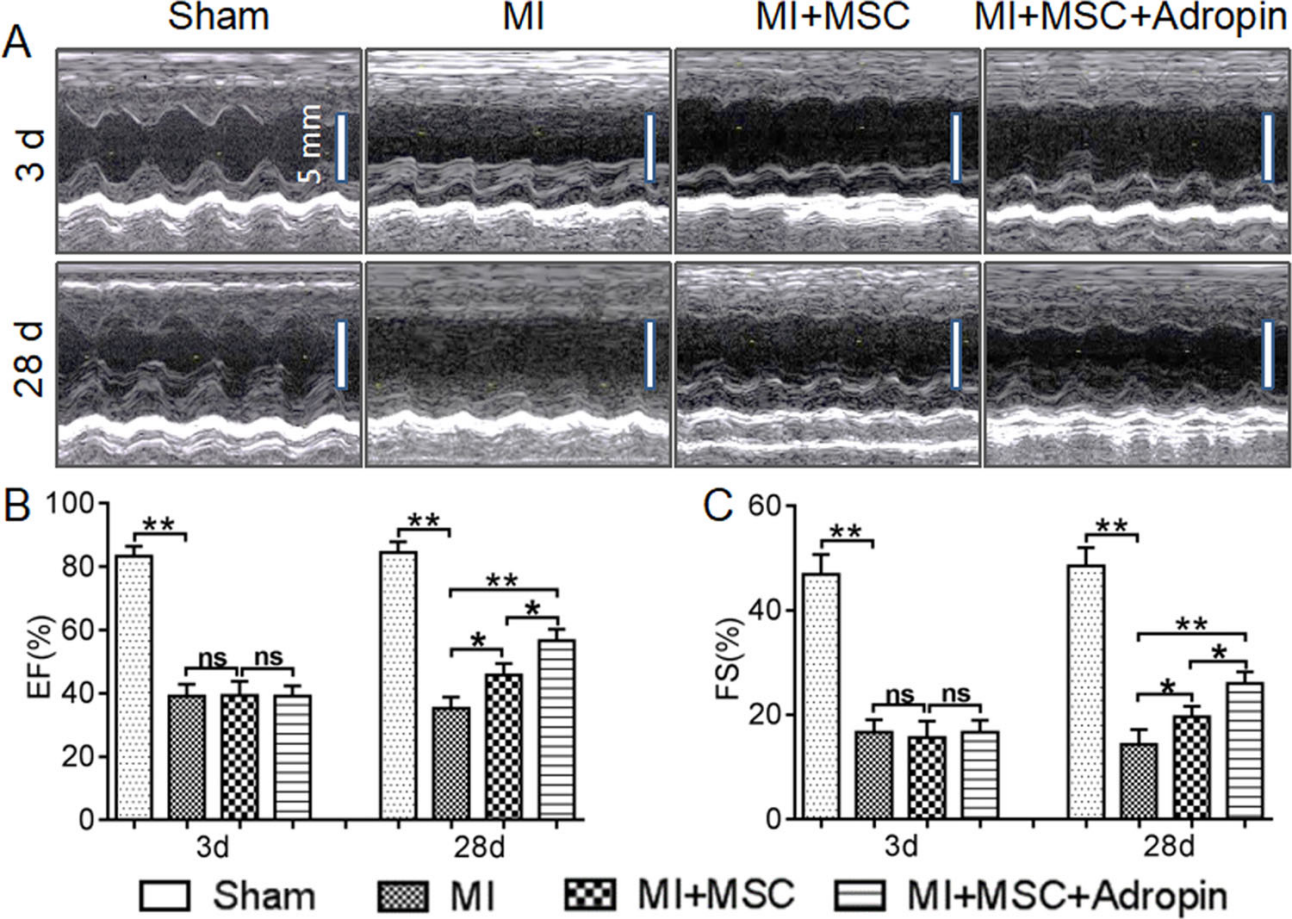
Improved cardiac function by adropin-based dual treatment through modifying both transplanted MSCs and host MI microenvironment in rats. The adropin-treated MSCs (modified “seed”) were implanted into the adropin-treated injured myocardium (modified “soil”), thus implementing dual protective effects of adropin. Representative echocardiograms in M-mode from four groups were shown in (**A**). Left ventricular EF and FS were shown in (**B**, **C**), respectively. MSCs Mesenchymal stem cells. MI Myocardial infarction. EF Ejection fraction. FS Fractional shortening. Data are expressed as mean ± SD, *n* = 8. *p < 0.05; **p < 0.01. NS No significance.

Echocardiography was performed to evaluate cardiac functional recovery after MI. At day 3 after cell transplantation, the left ventricular FS and EF between the MI + MSC + Adropin and MI + MSC groups were not statistically significant, though higher than those of the MI group (Fig. 2A–C). EF and FS in the Sham group were the highest among all the groups. At day 28, compared with the MI group, the MI + MSC and MI + MSC + Adropin groups showed a significant improvement in the left ventricular EF (35% ± 1% vs. 46% ± 1% and 57% ± 1%, *p* < 0.01, respectively) and FS (*p* < 0.01), and the MI + MSC + Adropin group (transplanted with adropin-pretreated MSCs and meanwhile treated with adropin, so called adropin-based dual treatment) had a higher EF and FS than the MI + MSC group (MSCs transplantation alone) (Fig. 2A–C), suggesting a beneficial impact of adropin-based dual treatment on therapeutic effects of transplanted MSCs in MI.

**Fig. 3:**
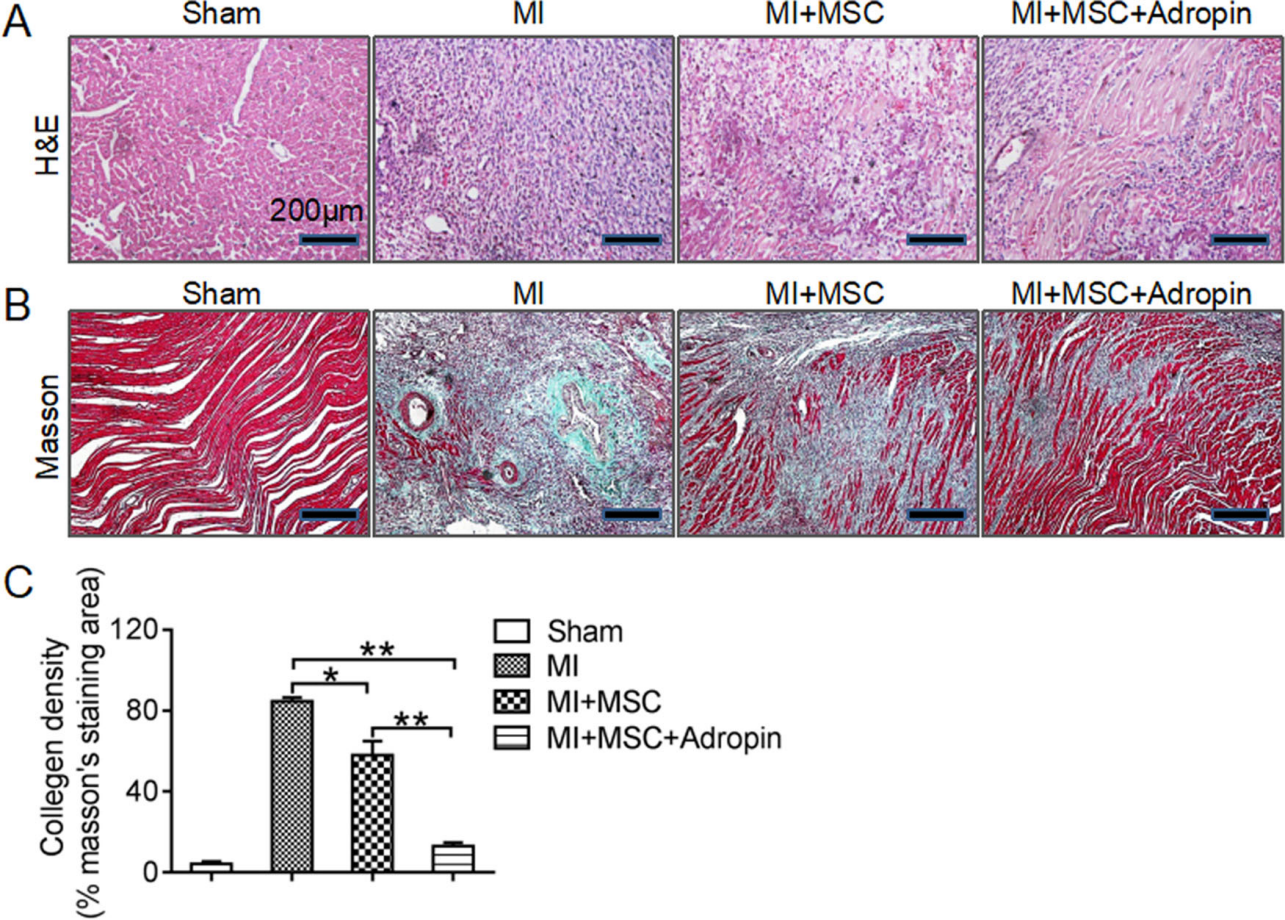
Alleviated cardiac remodeling by adropin-based dual treatment with MSCs transplantation in rat myocardial infarction. **A** H&E staining (original amplification: ×200). **B**, **C** Masson’s trichrome staining was used to assess myocardial fibrosis, and typical images were shown in (**B**) (original amplification: ×100). MSCs and MI, see Fig. 1. Data are expressed as mean ± SD, *n* = 8. *p < 0.05; **p < 0.01.

Consistent with the echocardiographic data, H&E staining showed that cardiac injury was significantly improved by adropin-based dual treatment when compared with MSCs transplantation alone (Fig. 3A). Masson’s trichrome staining revealed that adropin-based dual treatment more significantly reduced the myocardial fibrotic area than MSCs transplantation alone (Fig. 3B, C). These data demonstrate that adropin-induced dual protective effects enhance the repair of infarcted myocardium by improving cardiac remodeling and function, although no significant difference in survival rate was evident between the MI + MSC group and MI + MSC + Adropin group at day 28 (*p* = 0.407, by Log Rank test; *p* = 0.447, by Breslow test).

### Adropin-based dual treatment improves the inflammatory microenvironment and enhances paracrine secretion of transplanted MSCs in myocardial infarction

**Fig. 4:**
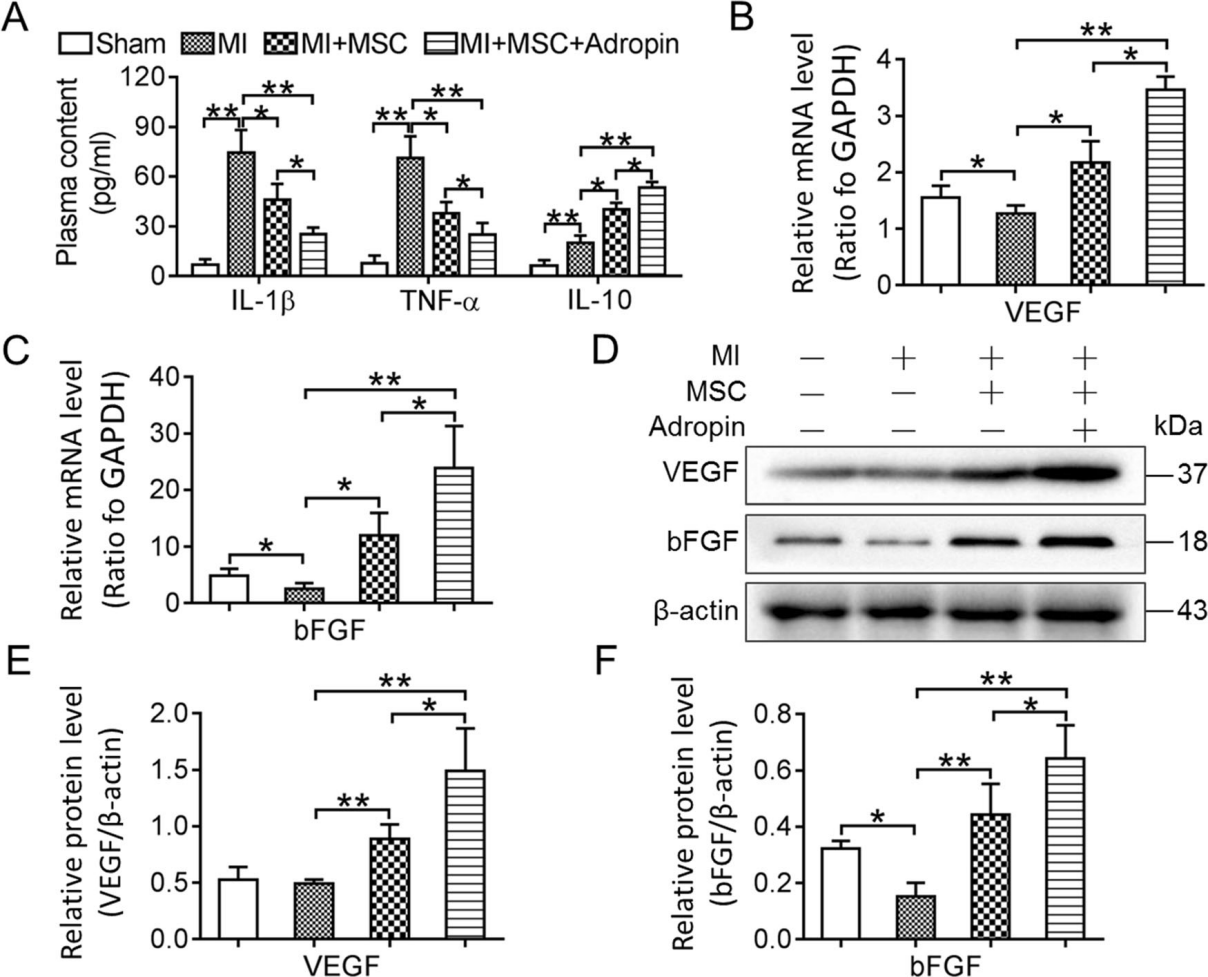
Modified inflammatory microenvironment and enhanced paracrine mechanism by adropin-based dual treatment with MSCs transplantation in rat myocardial infarction. **A** Plasma concentration of IL-1β, TNF-α, and IL-10 was detected by ELISA at day 3 after cell therapy to evaluate systemic inflammation induced by MI. **B**, **C** Real-time PCR was used to assess the gene expression of paracrine cytokines VEGF and bFGF in the infarcted myocardium at day 28 after cell therapy. **D**, **E**, **F** Western blotting was performed to evaluate the protein expression of VEGF and bFGF at day 28. IL-1β Interleukin-1β. TNF-α Tumor necrosis factor. IL-10 Interleukin-10. VEGF Vascular endothelial growth factor. bFGF Basic fibroblast growth factor. MSCs and MI, see Fig. 1. Data are expressed as mean ± SD, *n* = 8. *p < 0.05; **p < 0.01.

As mentioned above, the attenuated myocardial fibrosis and pathologic changes suggest that the cardiac microenvironment at the late stage of cell transplantation (at day 28) was improved by adropin-induced dual effects. We further evaluated the role of systemic inflammation, which is the most important characteristic of MI-induced hostile microenvironment at the early stage of cell transplantation (at day 3). The systemic plasma concentration of pro-inflammatory cytokines [interleukin (IL)-1β and tumor necrosis factor (TNF)-α] and anti-inflammatory cytokine (IL-10) was detected by ELISA (Fig. 4A). The Sham group had significantly lower concentrations of plasma IL-1β, TNF-α and IL-10, when compared with other groups (*p* < 0.01). Compared with the MI group, the MI + MSC and MI + MSC + Adropin groups showed a lower protein expression of IL-1β (*p* < 0.05) and TNF-α (*p* < 0.05), but a higher protein expression of IL-10 (*p* < 0.05). Compared with the MI + MSC group (MSCs transplantation alone), the MI + MSC + Adropin group (adropin-based dual treatment) had lower IL-1β and TNF-α, and higher IL-10.

**Fig. 5:**
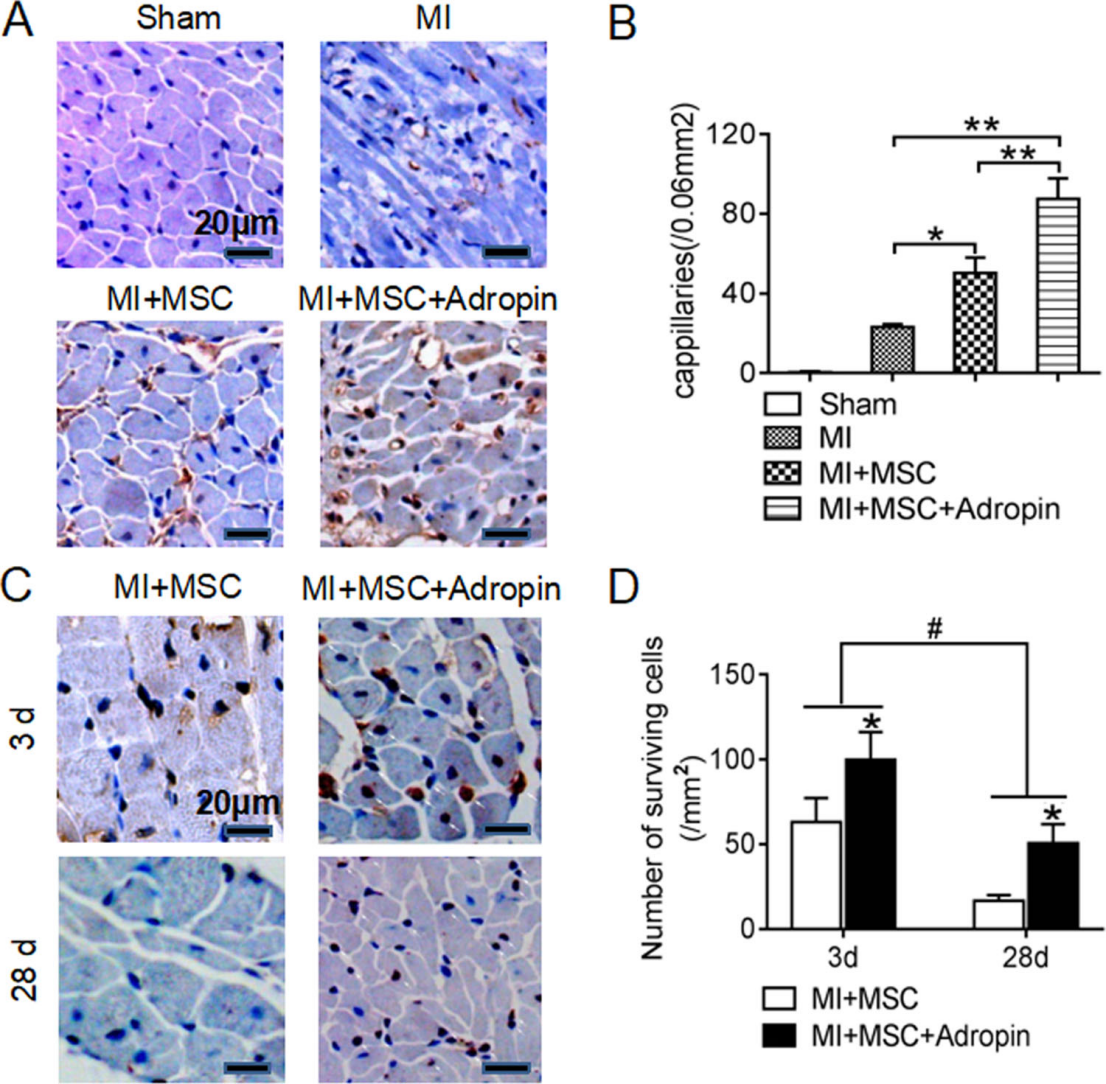
Increased neovascularization and number of surviving transplanted MSCs by adropin-based dual treatment in rat myocardial infarction. Immunohistochemical staining was performed to detect neovascularization and surviving implanted MSCs. **A**, **C** The positive staining for factor VIII-related antigen and surviving BrdU-positive implanted MSCs were brown by diaminobenzidine (DAB), and the nuclei were counterstained blue by hematoxylin (original amplification: ×200 in (**A**), ×400 in (**C**)). **B** Adropin-based dual treatment (the MI + MSC + Adropin group) had more neovascularization than MSCs transplantation alone (the MI + MSC group) at day 28 after cell therapy. **D** Adropin-based dual treatment conferred more surviving implanted MSCs than MSCs transplantation alone at the early (day 3) and late (day 28) stages after cell therapy. MSCs and MI, see Fig. 1. Data are expressed as mean ± SD, *n* = 8. *p < 0.05 and **p < 0.01 in (**B**). *p < 0.05 as compared with the MI + MSC group, and ^#^*p* < 0.05 in (**D**).

As important protective mechanisms for the improvement of cardiac microenvironment in stem cell therapy, paracrine factors [vascular endothelial growth factor (VEGF) and basic fibroblast growth factor (bFGF)] were further evaluated in heart tissues by RT-PCR and western blotting at day 28 after cell transplantation (Fig. 4B–F). Compared with the Sham group, the MI group showed a decrease in both mRNA and protein expression of bFGF and mRNA expression of VEGF. Both mRNA and protein expression of bFGF and VEGF were remarkably increased by MSCs transplantation alone or combined with adropin treatment. Actually, adropin-based dual treatment obviously accelerated the secretion of paracrine factors for a higher production of VEGF and bFGF in the MI + MSC + Adropin group than in the MI + MSC group. In accordance with increased secretion of paracrine cytokines (VEGF and bFGF) in MI microenvironment, adropin-based dual treatment augmented the effects of transplanted MSCs on neovascularization (Fig. 5A, B).

These data demonstrate that the adropin-based dual treatment with transplanted MSCs can produce a better effect on the secretion of local paracrine factors at a late stage and on the systemic inflammatory reaction at an early stage after cell therapy, which may exert beneficial effects on cardiac repair by neovascularization after MI.

### Adropin-based dual treatment promotes the survival of transplanted bone marrow MSCs

At day 3 and 28 after cell therapy, immunohistochemical staining was performed to determine whether the survival of transplanted stem cells may improve in response to adropin-based dual treatment (Fig. 5C, D). Surviving transplanted MSCs were identified as the BrdU-positive cells. In the MI + MSC + Adropin group (adropin-based dual treatment), at the early (day 3) and late (day 28) stages of cell therapy, the number of BrdU-positive cells was significantly higher than that of the MI + MSC group (MSCs transplantation alone) [at day 3, (100 ± 16)/mm^2^ vs. (63 ± 14)/mm^2^, *p* < 0.05; at day 28, (51 ± 11)/mm^2^ vs. (17 ± 3)/mm^2^, *p* < 0.05, respectively]. In both groups, the number of surviving transplanted cells at the late stage was less than that at the early stage (*p* < 0.05). The data indicate that adropin-based dual treatment promotes the survival of transplanted MSCs during the experiment.

## Discussion

In this study, using an in vivo rat MI model, we demonstrated that the transplantation of adropin-treated MSCs into adropin-treated infarcted myocardium, an adropin-based dual protective strategy, may enhance the therapeutic potential of MSCs to improve cardiac function by promoting the survival of transplanted cells and modifying host microenvironment through paracrine mechanism. Meanwhile, in vitro using a H_2_O_2_-induced MSCs apoptosis model, the antiapoptotic effects conferred by adropin are demonstrated to be involved in the increased transplanted cell survival via the pro-survival pathways.

The therapeutic effects of stem cell transplantation on cardiac repair depend on a number of influencing factors, including donor cells (the “seed”), host microenvironment (the “soil”), and the use of stem cells as a therapeutic remains challenging^18^. The weak “seed” and the hostile “soil” constitute a dual dilemma. Many efforts have been made to target either the “seed”^3,19,20^, or the “soil”^4,21,22^. However, all these may remain insufficient, clinically inaccessible, or even potentially detrimental^10,23,24^. Therefore, it is reasonable to perform a dual protective strategy targeting the dual dilemma (“seed” and “soil”). Moreover, bioactive substances may be an attractive strategy.

Adropin was identified in 2008 as a secreted protein, which is composed of 43 amino acids, and is produced by the proteolytic cleavage of 76 amino acid precursors^11^. It is expressed in the liver, brain, kidney medulla, muscles, gastrointestinal tract, coronary artery endothelial cells, and heart^11,25,26^. Furthermore, it is present in the circulatory system of animals and humans, and is negatively associated with the severity of coronary atherosclerosis^25–27^.

It has been demonstrated that adropin contributes to glucose and lipid homeostasis, and cardiovascular system functions^11,25^. The biological effects of adropin are mainly mediated through the activation of the orphan G protein-coupled receptor 19^26^. Adropin has a protective effect on the endothelial function and cardiomyocyte survival via the RISK pathway^12,14^. Since antiapoptosis by the activation of pro-survival cascades is an important strategy in optimized cell therapy^3,19,28^, in this study, adropin was used to produce dual protective effects.

This study demonstrated that in simulative in vitro cardiac hostile microenvironment induced by H_2_O_2_, adropin protected MSCs against apoptosis. Moreover, with the decrease in the protein expression of t-Akt, t-ERK1/2, BCL-2, and BCL-XL, adropin-induced antiapoptotic effects were blocked by the inhibitors of the RISK pathway. These data demonstrate that the RISK pathway may be involved in the adropin-induced in vitro cytoprotection of MSCs, which is consistent with our previous study of cardiomyocyte^14^.

On the other hand, the RISK pathway has also been well demonstrated to be involved in in vivo myocardial protection^13,17^ and the therapeutic effects of MSCs^3,29^. It has been shown that adropin protects endothelium by the RISK pathway^12^. In this study, adropin was used to in vivo modify cardiac microenvironment, together with the transplantation of adropin-treated MSCs, constituting an adropin-based dual treatment.

Our data showed that at day 28, compared with the MSCs therapy alone, the adropin-based dual treatment conferred more significant improvement in cardiac function and ventricular remodeling. However, the dual treatment did not improve the survival rate, which may be attributed to the insufficient cases, inadequate observation time, and limited effect of the intervention on mortality.

The present study showed that systemic inflammation, which is an important characteristic after acute MI, was attenuated by adropin-based dual treatment, indicating the improvement of cardiac microenvironment.

It has been well documented that the transplanted MSCs may improve cardiac function by secreting various paracrine cytokines, which may in turn promote the survival of MSCs and host cardiomyocytes by attenuating inflammation and apoptosis and accelerating microvessel regeneration to enhance regional blood flow^1–4^. The present study confirmed that in comparison with the MSCs therapy alone, adropin-based dual treatment increased the number of surviving BrdU-positive MSCs by 37% at day 3 after the transplantation and further salvaged surviving transplanted cells by 51% at the late stage (day 28). From 3 days to 28 days, 73% of transplanted cells were lost in the group receiving the MSCs therapy alone, but only 49% in the group receiving the adropin-based dual treatment. This evolving pattern was consistent with our previous study^4^.

Since the paracrine effects have been documented as a more important mechanism than myocardial regeneration in stem cell therapy^1–4^, in this study, we did not evaluate the transdifferentiation of transplanted MSCs to regenerate cardiomyocytes. Our data confirmed that at day 28 after stem cell therapy, MSCs significantly promoted the gene and protein expression in VEGF and bFGF, which were enhanced by adropin-based dual treatment through promoted capillary regeneration.

The plasma half-life of adropin has not been identified yet^25,26^. Because the half-lives of peptide hormones vary between 3 and 30 min, it is assumed that the half-life of adropin is as short as several minutes. We postulate that in this study, a single intravenous injection of adropin after ischemia may modulate cardiac microenvironment at the early stage of MI by suppressing inflammatory reaction, and adropin-pretreated MSCs may have stronger survival potential in the injured ischemic tissue, thus conferring the effects observed at day 28.

In this study, in consideration of potential indeterminate outcome targeting either “seed” or “soil” alone, we did not in vivo verify the respective effect of the transplantation of adropin-treated MSCs or an intravenous injection of adropin on cardiac outcome, which is a limitation. In spite of the potential effect of adropin administration itself on cardiac function, the promoted cardiac function by adropin-based dual treatment may not be a simple accumulation of respective effects of modified “seed” and “soil”, since the increased survival of transplanted cells by dual treatment-induced improvement of host microenvironment may at least partly contribute to the final promotion of cardiac function. However, it can not be determined whether the dual treatment actually generates synergistic effects in this study.

Even so, the present data demonstrate that adropin-induced dual protective effects may improve cardiac function and ventricular remodeling. Moreover, both the transplantation of MSCs and the administration of adropin were performed at 10 min after ischemia, thus favoring the clinical application and the early inhibition of ventricular remodeling process.

There are other limitations in this study. First, we evaluated the expression of VEGF and bFGF only at one time point. Second, the expression of apoptosis-related factors in infarcted myocardium was not evaluated, since their evolving pattern was consistent with the expression of systemic inflammation-related cytokines^4^. Third, importantly, we did not reproduce the main key in vitro findings in a hypoxia–reoxigenation model.

In conclusion, by using in vitro and in vivo models, we developed an adropin-based dual protective strategy to enhance therapeutic potential of stem cells by promoting transplanted cell survival (modifying “seed”) and improving host microenvironment (modifying “soil”) via the paracrine mechanism and the pro-survival pathways. This may be a novel optimized strategy for stem cell transplantation to repair infarcted myocardium.

## Supplementary information

supplementary information

Supplemental Figure Legends

suppl Figure 1

suppl figure 2

suppl figure 3

Suppl Figure 4

Suppl Figure 5
