# Supplementary material for: Adropin-based dual treatment enhances the therapeutic potential of mesenchymal stem cells in rat myocardial infarction
Source: Cell Death Dis. 2021 May 18;12(6):505. doi: 10.1038/s41419-021-03610-1 (PMC8131743; doi:10.1038/s41419-021-03610-1)
Supplement: Supplementary file 1 — supplementary information [file 41419_2021_3610_MOESM1_ESM.docx]

There are five supplementary figures total.
