# Supplementary material for: Adropin-based dual treatment enhances the therapeutic potential of mesenchymal stem cells in rat myocardial infarction
Source: Cell Death Dis. 2021 May 18;12(6):505. doi: 10.1038/s41419-021-03610-1 (PMC8131743; doi:10.1038/s41419-021-03610-1)
Supplement: Supplementary file 2 — Supplemental Figure Legends [file 41419_2021_3610_MOESM2_ESM.docx]

**Supplemental Figure Legends**

**Supplementary Figure 1.** **Determination of the best** **concentration and timing of hydrogen peroxide (H_2_O_2_) treatment for the** **establishment of cell apoptosis model.** Rat bone marrow mesenchymal stem cells (MSCs) were cultured for 1 hour with different final concentrations of H_2_O_2_ to determine the best concentration to establish cell apoptosis model. Cell viability (survival) was measured by cell counting kit-8 (CCK-8). Data were normalized against the control group (0 μmol/L of H_2_O_2_). The dose of 100 μmol/L with a significant decrease in survival cells was determined as the best concentration (A, B). Then, MSCs were furtherly cultured for 4 h to determine the best timing (C, D). According to the presence of significant apoptosis at 4 h culture, a final concentration of 100 umol/L H_2_O_2_ for 4 h was used to investigate the protective effect of adropin against MSCs damage. Representative images of cultured cells were shown in A and C (original amplification: × 200).

**Supplementary Figure 2. Determination of the appropriate dose of adropin treatment for cardioprotection.** The sham operation did not produce visible ischemic and necrotic area. The other groups showed no significant differences in AAR/LV. Representative pictures of tetrazolium chloride and Evan’s blue-stained heart sections from mice treated with different dose adropin were shown on the top of various groups in the left columns. The infarct areas are in pale color and the viable tissues are in red color. When compared with that of the I/R group, the myocardial infarct size (IS/AAR) was significantly reduced by an intravenous injection of adropin at a dose of 0.2 mg/kg in the Adropin-M group (45.8 ± 5.1 vs. 30.5 ± 5.2 %, *p*<0.05), but a lower dose of 0.1 mg/kg in the Adropin-L group failed to reduce IS and a higher dose of 0.4 mg/kg in the Adropin-H group did not achieve further IS reduction. This dose-effect study demonstrated that adropin at a dose of 0.2 mg/kg may be cardioprotective in rodents. Therefore, 0.2 mg/kg was used as an appropriate dose in the adropin-based dual treatment study. Data were presented as mean ± SD. ^*^ *p* <0.05 vs. I/R. ^#^ *p* <0.05 vs. Adropin-L. I/R, ischemia-reperfusion. IS, infarct size. AAR, area at risk. LV, left ventricle.

**Supplementary Figure 3. Surface markers, growth curve, and multiple differentiation of rat** **bone marrow mesenchymal stem cells (MSCs) under culture.** **A**: Cultured bone marrow cells had high CD44, CD90 and CD29 expression, and low CD45, CD34 and CD11b expression in cell surface, indicating the characteristics of MSCs. **B**: Growth curve of rat bone marrow MSCs under culture (from first to third passage) was shown. **C** (original amplification: × 200): Left: After 28 days of induction, chondrogenesis was indicated by a number of blue glycosaminoglycan synthesis with alcian blue staining. Middle: After 21 days of induction, osteogenesis was indicated by a mass of red calcium salinity accumulation with alizarin red staining. Right: After 14 days of induction, adipogenesis was indicated by the accumulation of neutral lipid vacuoles with oil red O staining. CD, cluster of differentiation. P, passage.

**Supplementary Figure 4. Determination of the labeling efficiency in BrdU-MSC.** BrdU-labeled MSC were about 87% positive in the MSC+PBS and MSC+Ad-M groups (n=6). BrdU, bromodeoxyuridine. MSC, bone marrow mesenchymal stem cells. PBS, phosphate buffer saline. Ad-M, moderate dose of adropin (25 ng/ml). DAPI, 4',6-diamidino-2-phenylindole.

**Supplementary Figure 5.** **The attenuated H_2_O_2_-induced apoptosis of bone marrow MSCs by adropin *in vitro*.** A: Determination of the best concentration of adropin: Adropin increased the survival of MSCs assessed by cell counting Kit-8 in a dose-dependent manner, and a higher dose (more than 25 ng/ml) of adropin cannot confer more protection. Therefore, the adropin dose of 25 ng/ml was used to test the effects on apoptosis. B, E: Early apoptosis was detected by annexin-V-FLUOS staining with FACS analysis, and typical graphs were shown. Apoptotic cells were shown in the area with high Annexin and low PI staining. C, F: FACS analysis showed the anti-apoptotic effects of adropin, which were bolcked by LY294002 and PD98059. D: The increased survival in MSCs by adropin treatment was also blocked by LY294002 and PD98059. H_2_O_2_, hydrogen peroxide. MSCs, mesenchymal stem cells. Ad-L, low dose of adropin (10 ng/ml). Ad-M, moderate dose of adropin (25 ng/ml). Ad-H, high dose of adropin (50 ng/ml). PI, propidium iodide. LY, LY294002, an inhibitor of phosphatidylinositol 3-kinase (PI3K)/Akt. PD, PD98059, an inhibitor of extracellular regulated protein kinases (ERK) 1/2. * *p*<0.05. ** *p*<0.01. NS, no significance.
