# Supplementary figures and images for: Adropin-based dual treatment enhances the therapeutic potential of mesenchymal stem cells in rat myocardial infarction
Source: Cell Death Dis. 2021 May 18;12(6):505. doi: 10.1038/s41419-021-03610-1 (PMC8131743; doi:10.1038/s41419-021-03610-1)

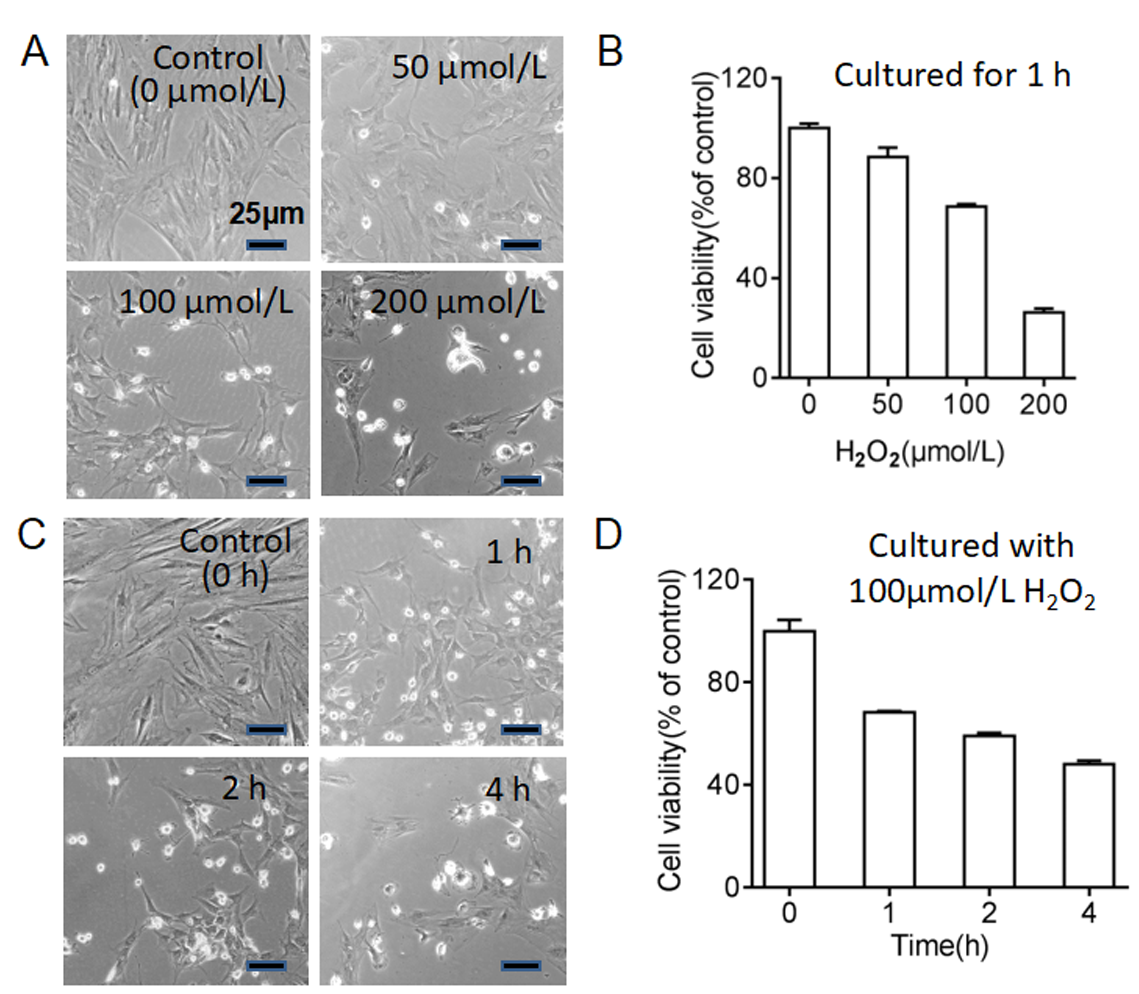

Supplement: Supplementary file 3 — suppl Figure 1 [file 41419_2021_3610_MOESM3_ESM.tif]

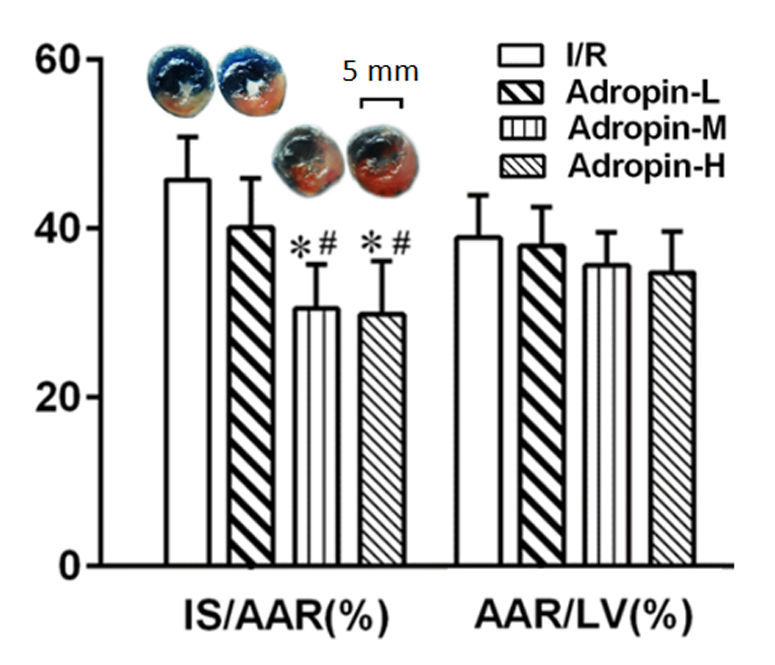

Supplement: Supplementary file 4 — suppl figure 2 [file 41419_2021_3610_MOESM4_ESM.tif]

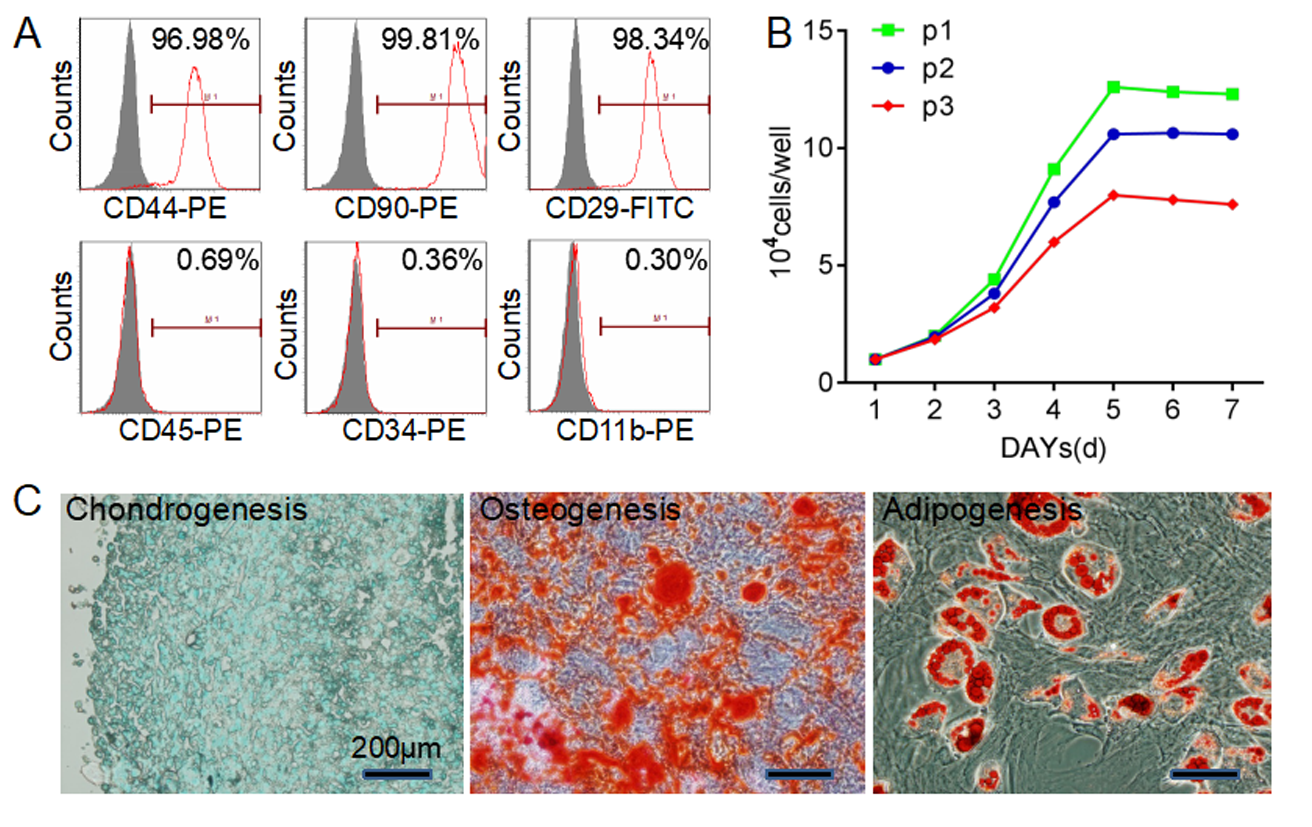

Supplement: Supplementary file 5 — suppl figure 3 [file 41419_2021_3610_MOESM5_ESM.tif]

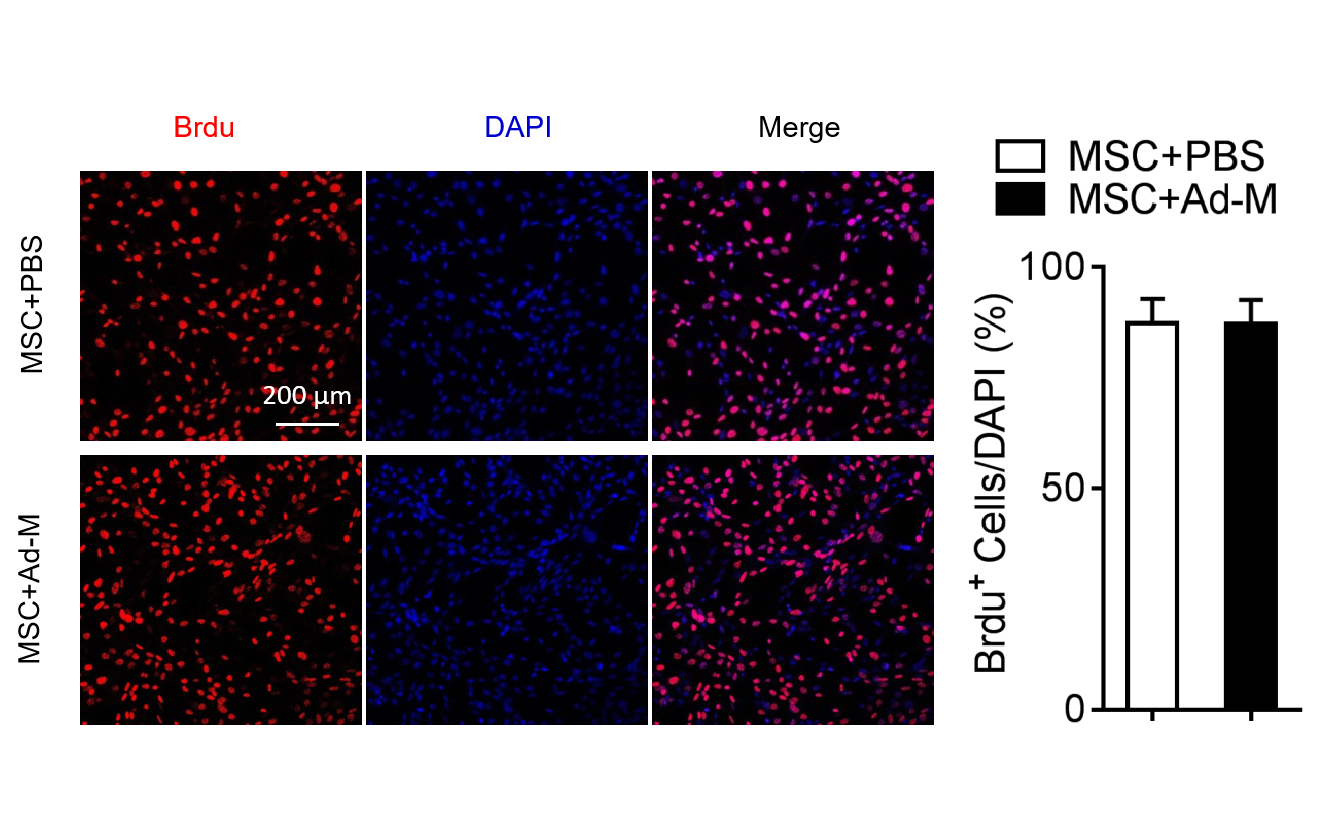

Supplement: Supplementary file 6 — Suppl Figure 4 [file 41419_2021_3610_MOESM6_ESM.tif]

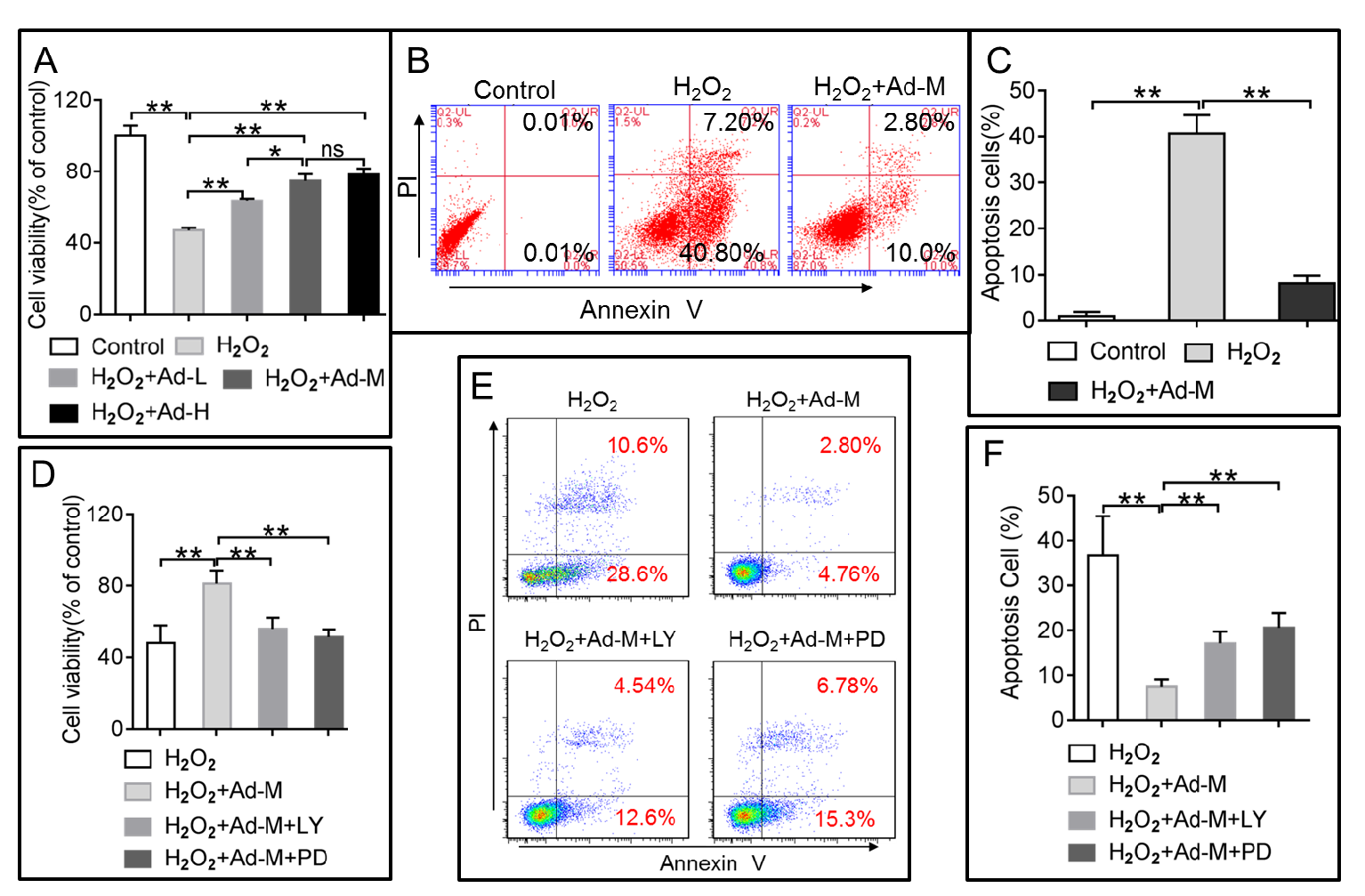

Supplement: Supplementary file 7 — Suppl Figure 5 [file 41419_2021_3610_MOESM7_ESM.tif]
